# Defining the decline: a glossary relevant to insect decline

**DOI:** 10.1093/jisesa/ieaf048

**Published:** 2025-05-13

**Authors:** Jessica Awad, Gagandeep Brar, Erin Cadwalader, DeShae Dillard, Lauren A Esposito, Elaine Evans, Christina Grozinger, Kelsey E Fisher, Akito Y Kawahara, Christian H Krupke, Andrea Lucky, Richard Mankin, Corrie S Moreau, Avalon C S Owens, Emily L Sandall, Katja C Seltmann, Jessica L Ware, Ross Winton

**Affiliations:** State Museum of Natural History Stuttgart, Stuttgart, Germany; Department of Biological Sciences, North Dakota State University, Fargo, ND, USA; State Museum of Natural History Stuttgart, Stuttgart, Germany; Entomological Society of America, Annapolis, MD, US; Department of Entomology, Michigan State University, East Lansing, MI, USA; Department of Entomology, California Academy of Sciences, San Francisco, CA, USA; Department of Entomology, University of Minnesota, Saint Paul, MN, USA; Huck Institutes of the Life Sciences, Pennsylvania State University, PA, USA; Department of Entomology, Connecticut Agricultural Experiment Station, New Haven, CT, USA; McGuire Center for Lepidoptera and Biodiversity, Florida Museum of Natural History, University of Florida, Gainesville, FL, USA; Department of Entomology, Purdue University, West Lafayette, IN, USA, USDA ARS Center for Medical, Agricultural, and Veterinary Entomology, Gainesville, FL, USA; McGuire Center for Lepidoptera and Biodiversity, Florida Museum of Natural History, University of Florida, Gainesville, FL, USA; USDA, ARS, CMAVE, Gainesville, FL, USA; Department of Entomology, Cornell University, Ithaca, NY, USA; The Rowland Institute at Harvard, Harvard University, Cambridge, MA, USA; Foreign Agricultural Service, U.S. Department of Agriculture, Washington, DC, USA; Foreign Agricultural Service, U.S. Department of Agriculture, Washington, DC, USA; American Museum of Natural History, NYC, NY, USA; Cheadle Center for Biodiversity and Ecological Restoration, University of California, Santa Barbara, Santa Barbara, CA, USA; Washington Department of Fish & Wildlife, Washington, DC, USA

**Keywords:** insect decline, biodiversity, definitions, entomology, conservation

## Abstract

Insects are declining in abundance and species richness, globally. This has broad implications for the ecology of our planet, many of which we are only beginning to understand. Comprehensive, large-scale efforts are urgently needed to quantify and mitigate insect biodiversity loss. Because there is broad interest in this topic from a range of scientists, policymakers, and the general public, we posit that such endeavors will be most effective with precise and standardized terms. The Entomological Society of America is the world’s largest association of professional entomologists and is ideally positioned to lead the way on this front. We provide here a glossary of definitions for biodiversity loss terminology. This can be used to enhance and clarify communication among entomologists and others with an interest in addressing the multiple overlapping research, policy, and outreach challenges surrounding this urgent issue.

Insects are declining at unprecedented rates globally (eg Hallmann et al. 2017, Stokstad 2018, Forister et al, 2019, Raven and Wagner 2021, Nath et al. 2023, Samu et al. 2023, Garcia-Gonzalez et al. 2024, see Wagner et al. 2021 for a review, and https://entogem.github.io/ for a synthesis of currently published papers). Despite many papers published on this topic within the last decade, limitations persist in baseline data for most insect groups regarding population structure, and the magnitude of potential declines. Although novel molecular approaches for generating biodiversity estimates (Liu et al. 2020) are helpful, there is an urgent need for comprehensive, large-scale efforts to understand and mitigate insect biodiversity loss.

In the interest of collectively approaching the issue of insect decline, in the fall of 2022, the Entomological Society of America (EntSoc) approved the creation of a Presidential Task Force on Insect Biodiversity, which was renewed for the 2023 to 2024 year. This Task Force was asked to examine how EntSoc practices may impact insect biodiversity (eg the environmental impacts of meetings and operational routines). As members of the Task Force, we have been working with EntSoc branches (5 within the US and 1 international), and sections (A) Systematics, Evolution and Biodiversity, (B) Medical and Veterinary Entomology, (C) Plant-Insect Ecosystems, and (D), Physiology, Biotechnology and Toxicology) to modify aspects of our activities with insect protection and conservation in mind. Further, we agreed to survey members of EntSoc to identify those working on insect decline (as broadly defined) with the intention to leverage expertise and further broaden collaborations, eg Saunders et al. (2020).

Drivers of insect decline are numerous, but most key drivers identified fit into the framework presented by Wilson and Fox (2021). Their study, adapted to include Kehoe et al. (2021), listed the following foci: habitat loss, climate change, pollution, and invasive species. However, we recognize that there are other drivers (eg see Fig. 1), and we hope to broaden our scope over time.

**Figure. 1.**
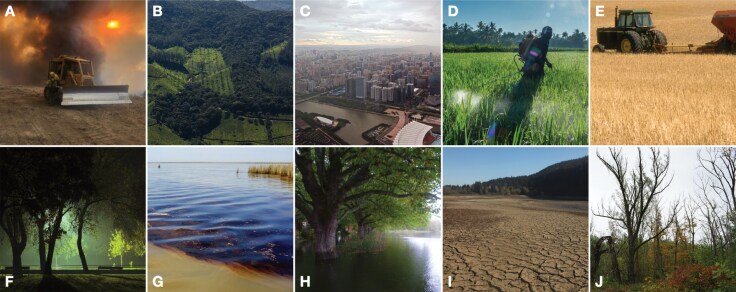
Top threats to insects include **A**) habitat loss, **B**) habitat fragmentation, **C**) urbanization, **D**) pesticide use, **E**) intensive agriculture, **F**) light pollution, **G**) chemical pollution, **H-I**) climate change, and **J**) invasive species. All images from Wikimedia Commons.

Insects are found in many terrestrial, aquatic, and underground ecosystems and throughout a vast array of habitats (eg Fig. 2). Entomologists work in fields, such as education, government, industry, museums, non-governmental organizations, small businesses, and universities. Because these fields differ in their approaches and terminology, it can be difficult to connect sources of insect biodiversity data. For example, someone monitoring larval mosquito populations for urban disease control may describe their work in very different ways than someone working in butterfly habitat conservation.

**Figure. 2.**
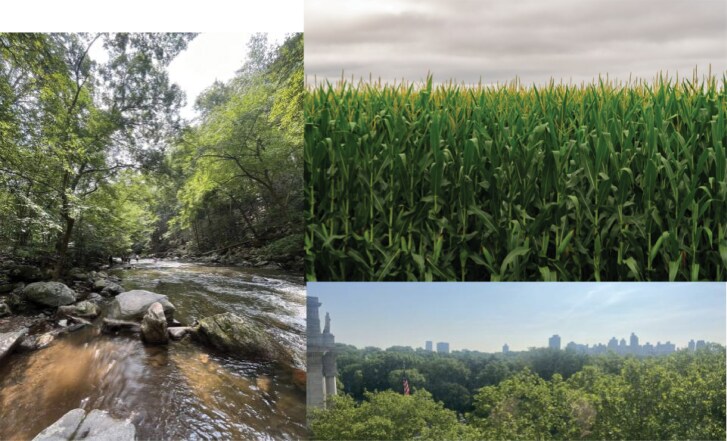
Examples of habitats in which insects are found: freshwater and terrestrial habitats (including but not limited to lotic systems, agricultural settings, and urban areas); aquatics image and urban image by Jessica Ware; agricultural image from Tony Webster from Minneapolis, MN, United States, CC BY-SA 2.0, via Wikimedia Commons.

There is a significant need for a common language when discussing insect decline (and insect population trends more generally; see Saunders et al. (2020) for discussion of when the word “decline” is appropriate to use when discussing insect biodiversity trends). While many entomologists may be largely familiar with most terms in this glossary, clarifying language and minimizing jargon can also aid in communicating effectively with a more diverse assemblage of partners by having a readily accessible list of terms to point to. This is a critical point—we know that the multi-factorial problem of insect decline will not be mitigated by entomologists alone. A list of definitions is given below to encourage a common vernacular when discussing insect declines. To help develop a coherent set of insect decline definitions, we discussed and constructed a taxonomy that can guide further efforts to assess, communicate, and mitigate insect biodiversity loss. This glossary of terms on the topic of insect declines can help advance discussion of this issue into the focus and forefront of biodiversity conservation efforts.

Conservation efforts for insects lag behind those for plants and many other animal groups, despite their economic and ecological importance (Samways 1993, Goulson 2019). Early insect conservation efforts often focused on charismatic species, primarily butterflies and other pollinators (Pyle 2012). Species that have not yet been identified cannot be the focus of insect conservation efforts; consequently, less well-known species are often more challenging to conserve because of the lack of taxonomic resolution and distribution data (see Fig. 3 for images of insect orders). In addition, although there has considerable discussion of interactions among plants, insect herbivores, and parasitoids (Miller and Dyer 2009), insects are often perceived only as pests rather than as active agents in natural and agricultural ecosystems, and this view has made it harder to obtain broad public support for insect conservation (Samways et al. 2020).

**Figure. 3.**
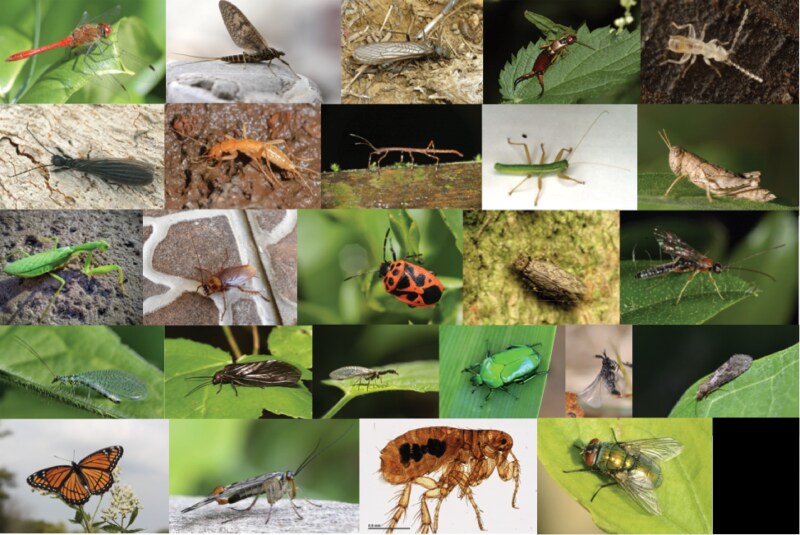
Examples of the breadth of insect biodiversity, with each order represented; image created using the following sources: Odonata: Huhu Uet, CC BY 3.0, via Wikimedia Commons Ephemeroptera: Richard Bartz, Munich aka Makro Freak, CC BY-SA 2.5 , via Wikimedia Commons Plecoptera: Bernard DUPONT from FRANCE, CC BY-SA 2.0 , via Wikimedia Commons Dermaptera: Fritz Geller-Grimm, CC BY-SA 2.5 , via Wikimedia Commons Zoraptera: Graham Montgomery, CC BY-SA 4.0 , via Wikimedia Commons Embioptera: CSIRO, CC BY 3.0 , via Wikimedia Commons Grylloblattodea: Marshal Hedin from San Diego, CC BY-SA 2.0 , via Wikimedia Commons Phasmatodea: Bernard DUPONT from FRANCE, CC BY-SA 2.0 , via Wikimedia Commons Mantophasmatodea: P.E. Bragg, CC BY-SA 3.0 , via Wikimedia Commons Orthoptera: Meva Rktn, CC BY 3.0 , via Wikimedia Commons Mantodea: Nwonwu Uchechukwu P, CC0, via Wikimedia Commons Blattodea: Emőke Dénes, CC BY-SA 4.0 , via Wikimedia Commons Hemiptera: Thomas Bresson, CC BY 2.0 , via Wikimedia Commons Psocodea: Katja Schulz from Washington, D. C., USA, CC BY 2.0 , via Wikimedia Commons Hymenoptera: Fritz Geller-Grimm, CC BY-SA 3.0 , via Wikimedia Commons Neuroptera: Mathias Krumbholz, CC BY-SA 3.0 , via Wikimedia Commons Megaloptera: Patrick Coin (Patrick Coin), CC BY-SA 2.5 , via Wikimedia Commons Raphidioptera: Frank Vassen from Brussels, Belgium, CC BY 2.0 , via Wikimedia Commons Coleoptera: Summerdrought, CC BY-SA 4.0 , via Wikimedia Commons Strepsiptera: Aiwok, CC BY-SA 3.0 , via Wikimedia Commons Trichoptera: Andrew C, CC BY 2.0 , via Wikimedia Commons Lepidoptera: USFWS Midwest, Public domain, via Wikimedia Commons Mecoptera: Richard Bartz, Munich aka Makro Freak Image:MFB.jpg, CC BY-SA 2.5 , via Wikimedia Commons Siphonaptera: Doc. RNDr. Josef Reischig, CSc., CC BY-SA 3.0 , via Wikimedia Commons Diptera: Polychronis Rempoulakis, CC BY-SA 4.0 , via Wikimedia Commons.

Overcoming these challenges is essential for a comprehensive understanding of the relevance of insect diversity in the development of methods to reduce harm from climate change and agricultural and natural ecosystems habitat losses. Although some actionable items have already been proposed to ease these threats (eg Forester et al. 2019, Kawahara et al. 2021), further scientific and public endeavors demand precise and standardized communication. By establishing clear definitions, we ensure that researchers, conservationists, policymakers, regulatory authorities, and the general public share a common language. This clarity reduces ambiguity and promotes effective collaboration.

Below is a list of definitions that we hope will encourage discussion using a common vernacular when discussing insect declines. These definitions will also serve as the basis for surveys that will be administered in the coming months to assess how EntSoc can collaboratively work toward the preservation of insect biodiversity. We curated this list to serve as a foundational lexicon essential for fostering a common understanding within the field of insect biodiversity study. Our methods in assembling this list were to (i) Choose terms to encapsulate key concepts and nuances crucial for comprehending, communicating, and advancing understanding in the realm of insect conservation and biodiversity, and (ii) provide definitions that offer a concise framework for comprehending the state of insect biodiversity, allowing researchers and conservationists to assess the health of ecosystems and make informed decisions. Terms and definitions were chosen and written by members of our Task Force spanning a wide variety of subfields within entomology. Understanding terms, such as “Endangered,” “Threatened,” and “Habitat Loss”, are pivotal for crafting effective conservation strategies. Also, we recognize the significance of ecological and environmental monitoring. Terms like “Insect Monitoring,” “Ecological Indicators,” and “Ecosystem Resilience” provide a foundation for designing monitoring programs, enabling the evaluation of biodiversity changes over time. Additionally, terms like “Dark Taxa” and “Taxonomic Impediment” acknowledge challenges in taxonomy, highlighting the need for increased research efforts.

These definitions align with international frameworks such as the Convention on Biological Diversity (United Nations Environment Programme, 1992), reflecting a global perspective in approaching insect conservation. This alignment facilitates collaboration and the exchange of knowledge on a worldwide scale. Finally, these definitions have educational value beyond the scientific community. They serve as a resource for students, educators, and the general public, fostering broader awareness and understanding of the critical role insects play in maintaining ecological balance.

Going forward, we aim to identify members working in this area across EntSoc Sections to creatively collaborate on solutions, work on infographics, videos and other public-facing products, and craft policy documents that may help both explain and begin to mitigate the steep declines in insect biodiversity reported globally.

## Proposed Insect Decline Biodiversity Term Definitions

### Abundance

A measure of the total number of individuals of a species or type present in a given area, in a given ecosystem, or within a particular habitat. Due to the cryptic nature of many insect species, exact counts of individuals (absolute abundances) can be difficult to obtain, but standardized sampling methods can show changes in abundances. In some cases, relative abundance, which examines how common or rare a species is compared to other species, is used to compare insect abundance changes.

### Aquatic Biomonitoring

The practice of assessing the quality of lentic and lotic water systems by making inferences based on the living taxa present in the water (eg microorganisms, macroinvertebrates including insects, and vertebrates).

### At-risk (imperiled) Species

Those species whose populations have declined significantly and may be in danger of extinction.

### Biodiversity

Also known as “Biological Diversity”; a measure of the genetic, ecological, and species variation in a specified location or on the planet. From the Convention on Biological Diversity (United Nations Environment Programme 1992), this is “the variability among living organisms from all sources including, inter alia, terrestrial, marine and other aquatic ecosystems and the ecological complexes of which they are part; this includes diversity within species, between species, and of ecosystems” (UN 1992).

### Biodiversity Loss

Decreasing number of living species; decline of biological diversity (Turnhout and Purvis 2020).

### Biomass

A measurement of the total mass or weight of the organisms present in a finite area or volume.

### Biosecurity

Methods implemented to prevent the invasion or spread of agents that would harm animals or plants, and/or spread disease.

### Climate Change

According to the United Nations (United Nations Environment Programme), “long-term shifts in temperatures and weather patterns”; can also refer to the umbrella term of “global warming.” While there are natural events that can result in these changes, the main driver of climate change is the burning of fossil fuels like coal, oil, and gas. Both annual weather and longer-scale climate changes are often grouped under the term climate change.

### Conservation

This word can mean different things depending on the context, but here we mean protection of natural resources, in this case insects, and prevention of human-driven extinction. Note that conserving or restoring a species involves working with the ecological community or community assemblage with which a species interacts.

### Conservation Biological Control

Changing the environment or control tactics to favor the abundance and activity of natural enemies to enhance their control of pests and pathogens (Naranjo et al. 2015, Stenberg et al. 2021, Lucatero and Philpott 2024). This is based on the use of natural enemies, as in traditional biological control, but focuses on resident taxa rather than mass-reared taxa from outside the region where control is taking place.

### Dark Taxa

Taxa for which little is known about species-level diagnostics, or cryptic taxa for which standard morphological methods have been unable to resolve taxonomies or taxa for which little study has been undertaken to resolve taxonomy.

### Ecological Filtering

Environmental processes and climatic or weather conditions that determine the species that survive in a given area.

### Ecological indicators

Aspects of an ecosystem that are monitored to assess human or other impacts, including the presence or absence of species (or abundance of a species relative to a particular threshold). Ecological indicators are often used to inform management practices.

### Ecological Integrity

An assessment of the structure, composition, function, and connectivity of an ecosystem as compared to reference ecosystems operating within the bounds of natural and historic disturbance regimes.

### Ecological Monitoring

Taking systematic, repeated measurements of environmental conditions with similar methods over time, particularly to enable long-term comparisons.

### Ecosystem

A dynamic complex of plant, animal, and microorganism communities and their non-living environment interacting as a functional unit.

### Ecosystem function

Ecosystem function includes the physicochemical and biological processes that occur within the ecosystem to maintain life.

### Ecosystem Resilience

The ability of an ecosystem to absorb change and return to equilibrium after a temporary disturbance.

### Ecosystem Services

The set of ecosystem functions provided by the natural environment that are directly linked to benefits to humans, although we can also consider this in the context of how organisms contribute to the health of a habitat.

### Ecotourism

Tourism that involves sustainable travel to natural habitats with an emphasis on observing, assisting, and improving local conservation efforts.

### eDNA

An abbreviation referring to “environmental DNA,” referring to genetic material present in environmental samples, such as soil, sediment, water, and air. Sampling environmental matrices is a useful method of detecting rare or cryptic species without the need to find individual target organisms and can be used to develop and compare biodiversity indices.

### Endangered

Any species that is in danger of extinction throughout all or a significant portion of its range. We note that the Endangered Species Act (Endangered Species Act 1973) does not include pest insects as endangered if they present “an overwhelming and overriding risk,” nor does the act consider insects to be endangered when considering only a portion of their geographical range.

### Environmental Monitoring

Activities to characterize environmental quality, typically including air quality, water quality, and soil health.

### Extinction

When there are no surviving individuals of a species.

### Extirpated

A local extinction; when a species is eliminated from a given geographic area, but is still found in other areas.

### Functional Diversity

The value and range of species’ traits that affect ecosystem functioning, such as the activities that species perform in a community or traits or niches of species that influence community interactions, such as diet breadth, parasitic behavior, or dispersal ability.

### Functional Extinction

When a species is unable to reproduce to produce a new generation, although individuals may survive for a time.

### Genetic Diversity

Variation in some or all of the genome among individuals, species or other taxonomic levels.

### Habitat

The place or type of site where an organism or population naturally occurs. Note that within this definition are subdefinitions for microclimate (climate in a restricted area that differs from the surrounding area), and microhabitat (a finite portion of the habitat with unique features that differentiate it from the surrounding area).

### Habitat Loss

The process by which a natural habitat becomes incapable of supporting its native species or is lost entirely. The elimination or alteration of the conditions necessary for insects and other invertebrates, vertebrates, and plants to survive in a pre-existing niche.

### Habitat Management

Management of land and its associated resources to maintain species in suitable habitats within their native geographic distribution.

### Habitat/Ecological Restoration

The practice of renewing and restoring degraded, damaged, or destroyed ecosystems and habitats in the environment by active human interruption and action.

### Insect Conservation

Protecting insects and preventing human-driven extinction; providing resources (eg habitat, hosts, mates) necessary to support insect populations.

### Insect Decline

Decrease in the number of species (biodiversity), total insects, and/or insect biomass.

### Insect Monitoring

Use of passive traps or sensing through electromagnetic, acoustic, visual, automated monitoring or biochemical means, active collecting, or lures to gain insight into insect activity and presence. In addition, environmental DNA (eDNA) and metabarcoding, which detect multiple species in a single DNA sample, are now being used as monitoring methods.

### Introduced Species

Non-native species that migrate or are transferred into a new environment, become established, thrive, and either are not harmful to the new environment or have an unknown impact.

### Invasive Species

Non-native species that are introduced to an environment, become established, thrive, and cause harm in the new environment. This term may include noxious and range-expanding species.

### Light Pollution

Illumination of the night sky and nocturnal habitats by human-made light sources, which may interfere with the activity of nocturnal organisms. Light pollution is a subjective term and safety lighting near a road or a sidewalk is not usually considered as light pollution.

### Metabarcoding

A DNA-based approach that identifies multiple species from a mixed sample (bulk DNA or eDNA) based on high-throughput sequencing (HTS) of a specific DNA marker or markers. The amount of DNA sequence data derived by HTS allows taxonomic identifiers to be rapidly assigned to many species present in a sample.

### Pesticide

Any substance or mixture of substances intended for preventing, destroying, repelling, or mitigating any pest, including insects and other arthropods, weeds, and fungal pathogens.

### Pollination Services

The process by which pollinators, like bees, other insects, birds, bats, and other animals, transfer pollen from one flower to another to achieve pollination, this includes, but is not limited to agriculturally important plants.

### Pollinators

Primarily insects (but also birds, bats, etc.), including any organisms that facilitate the fertilization of wild and domestic flowering plant species.

### Pollution

The introduction of materials or energy, broadly defined (including chemical contaminants and light), that contaminate natural habitats and cause harm, directly or indirectly, to biological organisms in a habitat.

### Protected Area

A geographically defined area that is designated or regulated and managed to achieve specific conservation objectives.

### Regenerative Agriculture

Farming in a way that prioritizes minimizing inputs and harnessing the ecology of systems to facilitate agricultural production.

### Soil Degradation

A decrease in soil quality at the physical, chemical, or biological level.

### Soil Quality

A measure of the ability of soil to support animal life, and maintain water and air quality based on its physical, chemical, and biological properties.

### Species Richness

A measure of the number of species in an area of given size.

### Sustainability

The ability to be maintained at a certain level or rate.

### Sustainable Agriculture

Farming in a way that will not compromise the ability of current and future generations to continue adequate agricultural production.

### Sustainable Use

The use of components of biological diversity in a way and at a rate that does not lead to the long-term decline of biological diversity, thereby maintaining its continuity through the present and into future generations.

### Taxonomic Impediment

A lack of taxonomic expertise and/or diagnostic resources, preventing the description and documentation of species diversity in a timely manner, before it goes extinct.

### Threatened Species

Any species that is likely to become an endangered species within the foreseeable future throughout all or a significant portion of its range.

### Water Quality

Descriptive characteristics of a body of water, including turbidity (concentration of suspended material in the water body, measured as the scattered or absorbed light at a given intensity), the quantity of bacteria, amount of salt in the water (salinity), the concentration of dissolved oxygen in the water, as well as measurements of pH, temperature, and the amount of pesticides and heavy metals.

## Conclusion

The primary goal of this list of insect decline-related terms is to facilitate an understanding of the breadth and impact of insect decline, and the threats driving those declines. Our hope is that improved communication about insect declines facilitates insect conservation actions. These terms are based on our current understanding of threats and available technologies. Future updates may be needed to stay abreast of technological advances and other developments.
